# Chronic Pulmonary Aspergillosis: Genomic Variant Analysis and Protein Dysfunction Susceptibility in a Brazilian Cohort

**DOI:** 10.3390/genes16060676

**Published:** 2025-05-30

**Authors:** Rafaela da Silva Mendes, Beatriz Martins Wolff, Mariana Ribeiro Costa Siemann, Yanca Gasparini Oliveira, Gleyson Francisco da Silva Carvalho, Lucas Liro Vieira, Eder Alencar Moura, Karina Marinho Nascimento, Lissandro de Sousa Rolim, Andre Nathan Costa, Marcello Mihailenko Chaves Magri, Vítor Falcão de Oliveira, Leslie Domenici Kulikowski

**Affiliations:** 1 Cytogenomics Laboratory, Departament of Pathology, Faculty of Medicine, University of São Paulo, São Paulo 05403-000, Brazillesliekulik@usp.br (L.D.K.); 2 Department of Pneumology, Hospital das Clínicas (HCFMUSP), University of São Paulo, São Paulo 05403-000, Brazil; 3 Department of Infectious and Parasitic Diseases, Hospital das Clínicas (HCFMUSP), University of São Paulo, São Paulo 05403-000, Brazil; marcello.magri@hc.fm.usp.br (M.M.C.M.); vitor.falcao@hc.fm.usp.br (V.F.d.O.)

**Keywords:** CPA, immune genetic, *Aspergillus*

## Abstract

Background/Objectives: Chronic pulmonary aspergillosis (CPA) is a debilitating condition often affecting immunocompetent patients with underlying structural lung diseases, particularly pulmonary tuberculosis. This study investigates single nucleotide variants (SNVs) in immunogenetic-related genes among a Brazilian cohort with CPA. Methods: Twelve patients with confirmed CPA, based on ESCMID/ERS criteria, were sequenced using custom multigenic panel sequencing. Variants were annotated, classified using ACMG guidelines, and analyzed for potential impact on protein interactions and immune pathways. Results: A set of SNVs in *CX3CR1*, *IL12B*, *IL4R*, *PTX3*, *CCR5*, and *IFNG* genes were classified as variants of uncertain significance (VUS), but protein–protein interaction analysis suggests a potential role in immune evasion and dysfunction. Conclusions: This is the first study to apply a custom multigenic panel for CPA susceptibility in a Brazilian cohort, contributing to future functional and clinical studies in fungal immunogenetics.

## 1. Introduction

Chronic pulmonary aspergillosis (CPA) is a progressive and potentially fatal fungal infection primarily caused by *Aspergillus* spp., an opportunistic fungus commonly found in the environment. Its clinical manifestation is influenced by both host immune status and the presence of pre-existing pulmonary sequelae [1,2,3]. Immunocompromised individuals—such as those undergoing solid organ or bone marrow transplantation—are particularly vulnerable to invasive aspergillosis [4]. However, CPA predominantly affects immunocompetent or non-neutropenic patients, especially those with chronic lung conditions such as tuberculosis sequelae [5,6].

CPA encompasses several clinical subtypes, including simple aspergilloma, chronic cavitary pulmonary aspergillosis, subacute invasive aspergillosis, *Aspergillus* nodules, and chronic fibrosing pulmonary aspergillosis. Diagnosis relies on a combination of radiological evidence, immunoprecipitation antibody titers, and clinical symptoms, which is especially critical in low-resource settings [7,8,9,10].

Despite treatment efforts, CPA is associated with significant morbidity and mortality, often progressing to pulmonary fibrosis and severely impacting quality of life [11,12]. Globally, an estimated 3 million individuals are affected by CPA annually, with Brazil reporting approximately 112,000–160,000 new cases per year and a 5-year mortality rate ranging from 38% to 85% [13].

Genetic predisposition may influence susceptibility to CPA. Single-nucleotide polymorphisms (SNPs) are common in the general population and can result from evolutionary adaptation, spontaneous mutations, or environmental exposure [14,15]. While not all genetic mutations result in pathogenic protein alterations, certain SNPs may affect immune response pathways. Genome-wide association studies (GWAS) have identified variants in immune-related genes, such as *PTX3*, associated with impaired neutrophil function and increased susceptibility to fungal infections [5,16,17].

Given the limited literature on host–pathogen genetic interactions in fungal infections, this study aims to investigate genomic variants in Brazilian patients diagnosed with CPA. Using next-generation sequencing (NGS) and American College of Medical Genetics (ACMG) classification guidelines, we identified and evaluated genetic variants that may contribute to susceptibility to CPA.

## 2. Materials and Methods

This study enrolled Brazilian patients receiving outpatient follow-up in the Infectious Diseases and Pulmonology departments at the Hospital das Clínicas, University of São Paulo (HC-FMUSP) during 2023, with a confirmed diagnosis of chronic pulmonary aspergillosis (CPA). All patients with CPA met the ESCMID/ERS diagnostic criteria [18], which include chest computerized tomography findings suggestive of aspergillosis, microbiological evidence of *Aspergillus* infection (microscopy, culture of respiratory samples, histology, or galactomannan), and/or serological evidence, and the exclusion of other possible diagnoses such as tuberculosis, malignancy, or similar conditions. Symptoms or radiological findings had to be present for at least 3 months [4].

CPA is a spectrum disease comprising five subtypes: simple aspergilloma, *Aspergillus* nodule, chronic cavitary pulmonary aspergillosis (CCPA), chronic fibrosing pulmonary aspergillosis (CFPA), and subacute invasive aspergillosis [18]. Simple aspergilloma involves a single pulmonary cavity with a fungal ball, typically associated with minimal or absent symptoms and stable imaging findings. Chronic cavitary pulmonary aspergillosis presents with one or more progressive cavities, significant symptoms, and radiological worsening. Subacute invasive aspergillosis affects mildly immunocompromised individuals and progresses more rapidly and aggressively than CCPA. The *Aspergillus* nodule consists of one or more nodules, with or without cavitation. Chronic fibrosing pulmonary aspergillosis is the advanced stage of CCPA, featuring fibrosis and major loss of lung function [18].

A total of 12 patients (n = 12) were included, of whom 8 were male (66.7%) and 4 were female (33.3%). All patients presented with post-tuberculosis pulmonary disease. The clinical subtypes observed in the cohort were: chronic cavitary pulmonary aspergillosis in 8 patients (66.7%), simple aspergilloma in 3 patients (25.0%), and chronic fibrosing pulmonary aspergillosis in 1 patient (8.3%). The exclusion criteria for the study were: immunosuppressive conditions (e.g., HIV infection, malignancies, prolonged corticosteroid administration, diabetes mellitus, and cirrhosis) and confirmed genetic diseases.

### 2.1. DNA Extraction

Peripheral blood (4 mL) was collected in EDTA-containing tubes. All participants signed informed consent forms (CAAE: 76601023.1.0000.0068). DNA was extracted at the Cytogenomics Laboratory, Department of Pathology, Faculty of Medicine, University of São Paulo, using the QIAamp DNA Blood Mini Kit (Qiagen^®^, Hilden, Germany), following the manufacturer’s protocol. A final volume of 30 µL was obtained for subsequent analyses, and DNA concentration and quality were measured using a Qubit fluorometer (Thermo Fisher Scientific^®^, Waltham, MA, USA).

### 2.2. Next-Generation Sequencing (NGS)

Exome libraries were prepared using the Illumina^®^ DNA Prep with Exome 2.0 Plus Enrichment kit (Illumina^®^, San Diego, CA, USA), according to the manufacturer’s instructions. Sequencing was performed using the NovaSeq 6000 System (Illumina^®^, San Diego, CA, USA). Raw data were generated in Variant Call Format (VCF) and annotated by comparison with the reference genome GRCh38/hg38 using bioinformatics tools.

### 2.3. Variant Filtering and Classification

A custom multigenic panel sequencing was developed, including genes previously reported to be associated with susceptibility to fungal infections. The panel included: *CCR5*, *CX3CR1*, *IFNG*, *IFNGR2*, *IL10*, *IL12A*, *IL12B*, *IL13*, *IL4*, *IL4R*, *CXCL8*, *CXCR1*, *CXCR2*, *MBL2*, *MIF*, *NOS3*, *PTX3*, and *ARNT2*. VCF files from each participant were analyzed using the Franklin by Genoox^®^ platform (https://franklin.genoox.com). Allele frequencies were obtained from the gnomAD v4.1.0 and ABraOM databases (Brazilian Online Archive of Mutations), as shown in Table 1.

Variants classified as single-nucleotide variants (SNVs) were evaluated using the American College of Medical Genetics and Genomics (ACMG) guidelines, considering allele frequency, in silico prediction tools, and mutation type. The criteria PM2_SUPP and BP4 were applied, and most variants were categorized as variants of uncertain significance (VUS)k, as shown in Table 2.

Additional analyses included computational modeling of amino acid substitutions and chemical dissimilarity to assess potential protein dysfunction. Protein–protein interaction networks and immune pathway enrichment were evaluated for implicated genes.

## 3. Results

### 3.1. Variant Analysis and Classification

Variant analysis was guided by the development of a virtual multigenic panel consisting of genes previously associated with susceptibility to fungal infections (Table 1). The selected genes were: *CCR5*, *CX3CR1*, *IFNG*, *IFNGR2*, *IL10*, *IL12A*, *IL12B*, *IL13*, *IL4*, *IL4R*, *CXCL8*, *CXCR1*, *CXCR2*, *MBL2*, *MIF*, *NOS3*, *PTX3*, and *ARNT2*. VCF files containing the sequencing data of each participant were uploaded to the Franklin by Genoox^®^ platform for variant annotation and classification. Global allele frequencies were obtained from gnomAD v4.1.0, while Brazilian-specific frequencies were retrieved from the ABraOM database.

### 3.2. ACMG Guidelines

Variants were evaluated using the ACMG criteria, including absence in population databases, in silico pathogenicity predictions, and variant type. Most SNVs were classified under the criteria PM2_SUPP + BP4 as variants of uncertain significance (VUS) (Table 2). Due to inconsistencies in in silico predictors, we further assessed the potential for structural and functional disruption by analyzing the chemical dissimilarity of substituted amino acids. This analysis supported the hypothesis of potential protein dysfunction. Additional evaluation using PPI networks revealed possible impacts on antifungal immune mechanisms.

### 3.3. Protein–Protein Interaction and Pathway Enrichment

**Figure 1:**
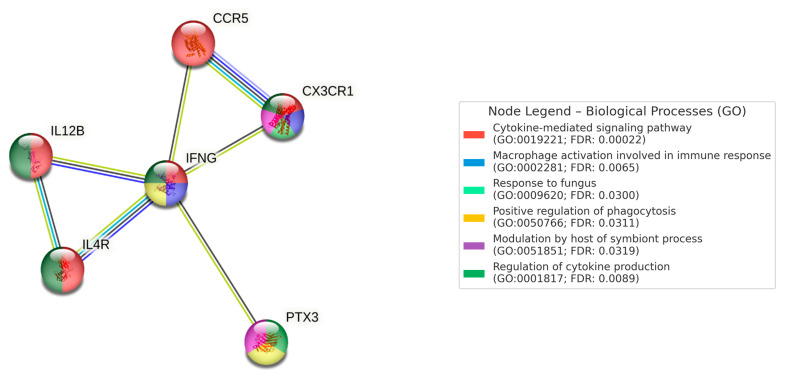
Number of nodes: 6, number of edges: 7, average node degree: 2.33, average local clustering coefficient: 0.867, expected number of edges: 1, PPI enrichment *p*-value: 6.27 × 10^−5^ (0.0000627).

PPI network analysis was performed using STRING v12.0, incorporating genes that showed VUS with potential immunological relevance (*PTX3*, *CX3CR1*, *IL4R*, *IL12B*, *IFNG*, *CCR5*). Significant enrichment was observed (PPI enrichment *p*-value < 0.05), suggesting interactions relevant to antifungal defense. Figure 1.

Biological processes identified included: cytokine-mediated signaling pathway (GO:0019221), macrophage activation in immune response (GO:0002281), response to fungal pathogens (GO:0009620), positive regulation of phagocytosis (GO:0050766), host modulation of symbiont processes (GO:0051851), and regulation of cytokine production (GO:0001817).

Relevant KEGG pathways included: cytokine–cytokine receptor interaction (FDR: 1.26 × 10^−6^), Th1 and Th2 cell differentiation (FDR: 0.00019), and the JAK-STAT signaling pathway (FDR: 0.00071).

## 4. Discussion

The primary clinical feature associated with progressive chronic *Aspergillus* spp. infection is structural damage caused by previous pulmonary diseases, such as tuberculosis. Interestingly, most affected individuals do not present significant alterations in leukocyte or lymphocyte lineages, which raises questions about immune system functionality in immunocompetent hosts. Effective immune responses require adequate signaling pathways. Although humoral immunity is not yet fully understood in fungal infections, it is clear that the absence of humoral signaling compromises the recruitment of cellular immune mechanisms. Pathogen recognition is mediated by soluble molecules that participate in immune cascades, including opsonization, phagocytic activation, and direct pathogen neutralization. This immunological synergy, particularly against Aspergillus conidia, requires the coordinated action of pattern recognition molecules and cellular immunity.

PTX3 is a soluble pattern recognition molecule that plays a vital role in recruiting macrophages and neutrophils to sites of inflammation. Its multifunctional nature includes modulating inflammation, tissue remodeling, and complement activation. Previous studies in mice have shown that knockout of *PTX3* is associated with increased susceptibility to fungal infections in non-neutropenic patients due to impaired conidial opsonization [19]. In our study, the variant rs138818541 in *PTX3* was identified in one patient (8.3%). Although classified as a variant of uncertain significance (VUS) by ACMG guidelines, emerging evidence suggests a deleterious impact of such variants [20]. Similarly, the rs555964469 variant in *CX3CR1* was also found in one patient. This chemokine receptor is predominantly expressed in natural killer cells, cytotoxic CD8+ T cells, macrophages, and monocytes. As leukocytes are essential in fungal clearance, defects in receptor function may compromise host defense. Studies like that of Lupiañez et al. (2020) [20] have shown that macrophages are key players in eliminating fungal pathogens, and alterations in their activation may contribute to CPA persistence. The pulmonary epithelium consists of alveolar epithelial cells type I and II (ATI and ATII). While ATII cells retain regenerative potential in adults, aging and chronic infections impair their functionality. The sustained recruitment of macrophages and inflammatory mediators, including profibrotic signals, can exacerbate CPA progression. Our cohort’s average age of 48 years may reflect environmental and age-related impacts on epithelial repair. Interestingly, the most frequently observed variant in our study was located in the *IL4R* gene (75% of patients). Although this gene is widely studied in allergic bronchopulmonary aspergillosis (ABPA), its role in CPA is less established. IL-4 is essential for Th2 cell differentiation, and abnormal expression of IL4R is implicated in Th2-skewed diseases. However, given the high allele frequency of most *IL4R* variants in the Brazilian population (ABraOM > 0.5), they were excluded from ACMG classification, except for the rare rs780006435 variant (allele frequency < 0.01), which met classification criteria. The presence of these variants suggests a molecular mechanism underlying increased vulnerability to CPA. While some in silico predictors did not classify them as deleterious, additional analyses of amino acid property changes suggested structural and functional alterations in the encoded proteins. Our study is limited by its small sample size due to the rarity of participants diagnosed with CPA without underlying conditions that cause some type of immunosuppression, which could contribute to biases in the study. The classification of variants takes into account several important factors for an appropriate conclusion. Although the classification is carried out, the scarcity of functional studies addressing the pathogen–host relationship and genetic regulation hinders a more robust analysis. Moreover, the Brazilian population’s genetic diversity complicates variant interpretation due to limited genome databases. Although we used ABraOM as a reference, it remains underpowered compared to European datasets. Nevertheless, this is the first study to apply a custom multigenic panel to a Brazilian CPA cohort. Our findings underscore the need for further functional studies and biomarker development, including quantification of PTX3 and MBL2 in clinical settings. Ultimately, integrating genetic insights into CPA management may improve diagnostic accuracy and therapeutic outcomes.

## 5. Conclusions

This exploratory study highlights the potential role of rare genomic variants in immune-related genes among patients with chronic pulmonary aspergillosis. Although the variants identified were classified as VUS, structural and network analyses indicate possible impacts on protein function and antifungal immune responses. This research represents the first application of a multigenic panel in a Brazilian cohort with CPA and emphasizes the importance of integrating genomic data into the investigation of host susceptibility to fungal diseases. Future studies with larger cohorts and functional validation are essential to better understand the molecular basis of CPA and to advance personalized approaches for its diagnosis and treatment.

## Figures and Tables

**Table 1:** Virtual multigenic panel. Column 1: name of genes. Column 2: Reference of literature Column 3: SNP ID. Column 4: Related to infections.

Gene	Reference	SNP ID	Related to Infections
*MBL*	PMID: 37558798	rs11003125	yes
*IL4*	PMID: 26667837	rs2243248	yes
*IL4R*	PMID: 27708669	rs3024656	yes
*IL8*	PMID: 26667837	rs2227307	yes
*CXCR1*	PMID: 26667837	rs2234671	yes
*CXCR2*	PMID: 26667837	rs1126580	yes
*PTX3*	PMID: 33240991	rs3816527	yes
*CX3CR1*	PMID: 31964743	rs9823718; rs7631529	yes
*MIF*	PMID: 36166743	NOT INFORMED	yes
*CCR5*	PMID: 26667837	rs1799987; rs2734648	yes
*IFNyR1*	PMID: 37327531	rs2234711	yes
*IFNy*	PMID: 26667837	rs2069705	yes
*IL13*	PMID: 26667837	rs1800925	yes
*IL12B*	PMID: 26667837	rs3212227C	yes
*NOS3*	PMID: 38407762	rs1549758	yes
*ARNT2*	PMID: 31964743	rs1374213	yes

**Table 2:** Variant classification. Column 1: Name of gene. Column 2: SNP ID. Column 3: Exon number. Column 4: Zygosity. Column 5: type of molecular alteration found. Column 6: ABraOm Allele frequency. Column 7: Global allele frequency. Column 8: Level of mutation damage. Column 9: Prediction of the effect of substitutions between amino acids based on chemical properties. Column 10: Classification according to ACMG.

Gene	SNP ID	HGVS	Exon	Zygosity	Alteration	ABrAom Allele Frequency	Global Allele Frequency	Prediction Tools	Grantham Distance	ACMG Classification
*CX3CR1*	rs555964469	NM_001337.4:c.457G>A	2	het	missense	0	0.00009788	0.303	Conservative (29)	VUS
*IL12B*	rs1245834629	NM_002187.3:c.835G>A	6	het	missense	0	0.000001859	0.0580	Moderately conservative (56)	VUS
*IL4R*	rs780006435	NM_000418.4:c.559G>A	7	het	missense	0	0.00001363	0.0850	Moderately conservative (58)	VUS
*PTX3*	rs138818541	NM_002852.4:c.1079G>A	3	het	missense	0.002135	0.0007912	0.237	Conservative (43)	VUS
*CCR5*	rs1800940	NM_001394783.1:c.180G>T	2	het	missense	0.002135	0.001624	0.154	Moderately radical (110)	VUS
*IFNG*	rs76012457	NM_000619.3:c.161G>A	2	het	missense	0.000427	0.00001305	0.0340	Moderately conservative (94)	VUS

## Data Availability

The data presented in this study are available on request from the corresponding author due to privacy.
